# Micronutrients deficiencies and its correlation with the soil-transmitted helminthic infections among children and non-pregnant women in Nepal: findings from Nepal national micronutrient status survey

**DOI:** 10.1038/s41598-022-24634-3

**Published:** 2022-12-24

**Authors:** Suresh Mehata, Kedar Raj Parajuli, Binod Rayamajhee, Uday Narayan Yadav, Ranju Kumari Mehta, Dipendra Raman Singh

**Affiliations:** 1 Ministry of Health, Province 1, Biratnagar, Nepal; 2 Nutrition Section, Family Welfare Division, Department of Health Services, Kathmandu, Nepal; 3 School of Optometry and Vision Science, Faculty of Medicine and Health, UNSW, Sydney, Australia; 4 Department of Infection and Immunology, Kathmandu Research Institute for Biological Sciences, Lalitpur, Nepal; 5 National Centre for Epidemiology and Population Health Research, School of Population Health, The Australian National University, Canberra, Australia; 6 Centre for Primary Health Care and Equity, UNSW, Sydney, Australia; 7 Center for Research Policy and Implementation, Morang, Nepal; 8 Little Buddha College of Health Sciences, Kathmandu, Nepal; 9 Department of Health Services, Kathmandu, Nepal

**Keywords:** Microbiology, Diseases, Health care

## Abstract

Despite having a high risk of soil-transmitted helminths (STHs) infection, no national level study has been conducted to assess incidence and correlates of STHs in Nepal. Thus, we hypothesized that micronutrients and social status are linked with incidence of STHs infection among 6–59 months children and 15–49 years non-pregnant women in Nepal and Kato-Katz technique was adopted to measure the type and intensity of STHs infections using fresh stool specimens and venous blood was used to examine micronutrients biomarkers. Anthropometric measurements such as height and weight, sociodemographic and health status were determined using structured questionnaire. Logistic regression was used to assess unadjusted and adjusted odds ratio (AOR) and the 95% CIs of ORs. The bivariate association of STHs was assessed with the covariates variables. Overall, 12% children and 19% non-pregnant women had STHs infection; *A. lumbricoides* was the predominant helminth in both study participants. In multivariate model; age, ethnicity, anaemia and zinc deficiency were associated with STHs infections in children. Similarly, higher odds of STHs occurrence was observed among non-pregnant women with vitamin A deficiency. Findings from this study suggest that high-risk population, with a focus on those of lower socioeconomic status should be on priority of deworming program, nutrition intervention, and mass administration of preventive chemotherapy and sanitation champions supplement to reduce the STHs infections in Nepal.

## Introduction

Widely distributed chronic infections caused by soil transmitted helminths (STHs) are one of the major public health concerns among people living in low and middle-income countries, particularly affecting the poorest and most deprived communities^1^. Almost 1.5 billion people are infected with STHs worldwide which includes infection of roundworm (*Ascaris lumbricoides*), whipworm (*Trichuris trichiura*), and hookworms (*Necator americanus* and *Ancylostoma duodenale*)^2^. World Health Organization (WHO) estimates that over 568 million school-going children and more than 267 million preschool children are at risk of STHs infections and are in urgent need of preventive interventions^3^.

Soil-transmitted helminth infections is a major public health concern among young children and studies have shown that STHs infections greatly impair their cognitive development and nutritional status^4,5^. The STHs usually do not present any symptoms unless there is a heavier infection which results into malnutrition, mal-absorption, abdominal pain, anemia, diarrhea, physical and mental growth retardation leading to important health consequences^2,6,7^. Severe STHs infections can cause even cognitive deficit and educational loss in school-aged children^8,9^. The risk of STHs infection is high in the regions with poor personal and environmental sanitation practices, eating and drinking of contaminated food and water, unhygienic toilet systems, handling or playing of infested soils and interaction with all these factors.

Importantly, the global burden of STHs infections is also linked with micronutrient such as vitamins, iron, and folate deficiencies and anemia which leads to reduced cognitive function and work capacity^10^. Malnutrition and anemia are associated with the severity of infections among the infected individuals, especially among pregnant women and school-age children. The synergistic existence of anemia, malnutrition and helminthiasis exert a negative impact on healthy growth of the infected individual^11^. Different types of STHs infection heavily affect nutritional status in different ways ranging from nutrient absorption to degree of mucosal damage^12^. Inadequate intake of Zn, Fe, and vitamin A predisposes individuals to helminth infections leading to nutritional deficiencies, thereby assisting helminth survival and severe infection^13^. Helminths feed on host tissues including blood which can cause loss of protein and iron of host. In addition, helminths may contest for vitamin A in the host intestine and some STHs cause loss of appetite whereas *T. trichiura* causes diarrhea and dysentery^14^. Low immune response due to malnutrition may increase susceptibility to STHs infections, therefore, nutritional supplementation is considered as a feasible means of controlling the morbidity due to STHs infections^15^.

The STHs infection is most prevalent among young children and women of reproductive age in the rural region of Nepal^16–18^ as a long standing health burden of the country^19^. In this light, a systemic review from Nepal found the pooled prevalence of 1.53% for hookworm, 4.23% for roundworm, and 2.89% for whip worm after 2004^20^. The prevalence of STHs was reported to be much higher in rural areas (24.1%) compared to urban (18.7%), where the higher burden was influenced by various factors including poor sanitary habits, inadequate drinking water access, insufficient hand washing habit and walking barefoot in farming fields^21–23^. In addition, multiple micronutrients deficiency such as iron, vitamin A, iodine, folate, zinc and the condition of anemia has remained as a hidden hunger specifically, among young children of rural Nepal which has put them at a higher risk of severe STHs infections^24,25^. Pre-school-age children (PSAC) are also heavily infected by different helminths and acute complications are often reported such as *Ascaris lumbricoides* infection may lead to bowel obstruction, duodenal ulcer, hepatic abscesses and appendicitis^26^.

STHs, one of the neglected tropical disease (NTDs) is an important target of NTDs 2021–2030, a road map setup by the United Nations and the WHO to cover 90% of people in disease intervention programs by 2030^27^. This highlights the importance of STHs control programs within the framework of universal health coverage to reduce the global burden promoting “leaving no one behind”. With this regard, Government of Nepal is working with multi stakeholders to tackle STHs infections through National Deworming Program, however diverse challenges exist due to unavailability of up-to-date national level epidemiological data to inform policy decisions and guide ongoing National Deworming and WASH (Water, Sanitation and Hygiene) programs in multiple provinces. To address this gap, our study aimed to assess the prevalence of STHs and its association with micronutrients deficiencies among children aged 6–59 months and non-pregnant women aged 15–49 years in Nepal, using nationally representative data.

## Materials and methods

### Study population and data sources

We used cross-sectional data from a nationally representative Nepal National Micronutrient Status Survey 2016 (NNMSS-2016). A detail methodology has been presented elsewhere^28,29^. The NNMSS-2016 study was conducted to provide up to date status on the basic health and nutrition status of 6–59 months old children and 20–49 years old non-pregnant women along with the associated demographic statistics. Stratified multistage cluster sampling without replacement approach was applied in the study in order to obtain the nationally representative estimates. Three geographical regions (Terai, Hill, and Mountain) and five development zones (Eastern, Central, Western, Mid-western, and Far western) were included hence the country was divided into 15 strata for this study (eastern mountain, eastern hills, eastern terai, central mountain, central hills, central terai, western mountain, western hills, western terai, mid-western mountain, mid-western hills, mid-western terai, far-western mountain, far-western hills and far-western terai). A minimum size of clusters was considered of 100 households and in case of wards having less than 100 households were merged with the adjacent wards whereas the clusters with more than 300 households were divided into the sub wards of 100 households. A total of 180 clusters (75 from the Terai and Hills each, and 30 clusters from the Mountains) were selected from the 15 strata considering the population proportion size^30^ where clusters (wards) were used as the primary sampling units (PSUs). A detail table of clusters is presented in the Table 1. A total of 24 households were selected from each cluster (24 × 180 = 4320) using a systematic random sampling. Cluster wise map was created by data collection team with the help of key informants such as teachers, female community health volunteers, etc. Subsequently, 24 households were selected systematically by the cluster supervisors prior the visit of field enumerators. A total of 4309 households were interviewed with the response rate of 99.7% (11 households were not available at the time of survey or refused to participate in this survey). Among the sampled households a total of 1728 children aged 6–59 months were eligible for interview and a total of 1709 children aged 6–59 months were interviewed with the response rate of 98.9% (1709/1728). Similarly, among 2160 non-pregnant women of aged 15–49 years were eligible for this study and 2144 were interviewed with the response rate 99.3% (2129/2144). Further, the participants with the missing values for STHs status among 6–59 months children (n = 174) and non-pregnant women (n = 117) were excluded from analysis. The final sample used in this analysis were 1535 children aged 6–59 months (89.8%) and 2012 non pregnant women (94.5%). Moreover, the non-response sample do not possess any systematic biases.

**Table 1:** Strata-wise distribution of sampling clusters.

Strata	Number of selected clusters	Remarks
Eastern Mountains, Central Mountains, Western Mountains, Mid-Western Mountains, Far-Western Mountains	30	6 per Strata
Eastern Hills, Central Hills, Western Hills, Mid-Western Hills, Far-Western Hills	75	15 per Strata
Eastern Terai, Central Terai, Western Terai, Mid-Western Terai, Far-Western Terai	75	15 per Strata

### Data collection

The data collection was conducted in April–June 2016 using structure questionnaire. A total of 14 teams were deployed for this study. Each team was composed of a field supervisor, enumerators, and phlebotomist, and laboratory technicians altogether of 13 members to collect all the required data including the blood sample. Phlebotomists have collected blood (in EDTA and plain vials as per the standard protocol). Pathologists have collected urine and stool samples from the study participants. Non-fasted blood samples were collected since fasting was not possible in the survey. Pathologists have collected stool samples within 24 h of the interview. Further, the samples were shipped to the Kathmandu considering the standard protocol (maintaining the 2–8 °C cold chain), where they were stored at − 80 °C at the National Public Health Laboratory (NPHL) for further processing.

### Anthropometry

Height/length-measuring board (Short-Board) was used to assess height/length and an electronic SECA digital scale (UNICEF Electronic Scale/Uniscale) was used to measure the weight of all recruited participants. The scale allows for the weighing of very young children through an automatic mother–child adjustment that eliminates the mother's weight while she is standing on the scale with her baby. After the measurement, the results of anthropometric measurements were immediately recorded on the respective questionnaire sheets^31^. In order to calculate anthropometric (*z* score) for children under five years the WHO Anthro survey analyzer version 1.0.3 was used. Body Mass Index (BMI) was calculated as body weight in kilogram divided by height in miters square (weight in kg/height in m^2^) of 20–49 years non-pregnant women.

### Biological specimens

STHs infections among the enrolled children (aged 6–59 months) and non-pregnant women (aged 15–49 years) was assessed. The STHs infection caused by *A lumbricoides*, *Trichuris trichura* and hookworms was examined using the Kato-katz technique. Kato-Katz (KK) technique was adopted by the laboratory technicians to measure the type and intensity of STHs infections using fresh stool specimens. Fresh stool specimens were collected from participants next day morning of interview date. All the participants were instructed about the stool specimen collection procedure. Collected stool specimens were stored in the cold boxes, and later transferred to pathologists for testing of STHs. One gram of collected stool specimen was used to perform KK method in the study field and within 24 h of stool specimen collection, the pathologist prepared duplicate slide for microscopy of each specimen and counted helminths eggs using a portable microscope^32^. In addition, stool samples were used to detect the *Helicobacter* *pylori* antigen^33^.

### Biomarkers of nutritional status

Photometric method using HEMOCUE Hb 301 analyzer was used to measure the hemoglobin (Hb). Hb < 11 g/dL were considered as the anemia for children aged 6–59 months whereas Hb < 12.0 g/dL were considered as anemia for non-pregnant women aged 15–49 years.

The sandwiched ELISA technique was used for the serum ferritin, Alpha-1 acid glycoprotein (AGP), C-reactive protein (CRP) and Retinol Binding Protein (RBP) at the VitMin Lab, Willstaett, Germany. Micronutrient parameters were adjusted according to the inflammation status. The antibodies used for this measurement were as follows: Capture antibodies: ferritin (Code A0133, Dako), RBP (Code A0040, Dako), and CRP (Code A0073, Dako Denmark). Detection antibodies: antiferritin-horseradish peroxidase (HRP) (Code P0145, Dako), anti-RBP-HRP (Code P0304, Dako), and anti-CRP-HRP (Code P0227, Dako). Iron deficiency was defined as if adjusted ferritin < 15.0 μg/L^34^.

Serum retinol was measured by HPLC is the WHO recommended indicator for vitamin A status. All the sample collected for this study were assessed to measure retinol-binding protein (RBP) using a sandwich ELISA at the VitMin Laboratory in Germany^35^. Vitamin A deficiency was defined as if RBP < 0.64 μmol/L^35^.

RBC folate was measured at Peking University, Institute of Reproductive and Child Health Laboratory (Beijing, China). Gold standard microbiological method for RBC folate concentration in RBC hemolysate was used following the protocol of O’Broin and Kelleher^36^. Risk of folate deficiency was classified as if RBC folate < 305.0 nmol/L based on risk of macrocytic anemia. We classified zinc deficiency as zinc < 66.0 μg/dL for nonfasted, morning samples (i.e. before 12 pm) and < 59.0 μg/dL for non-fasted, afternoon samples (i.e. after 12 pm) for non-pregnant 15–49 years women while we classified zinc deficiency as < 65.0 μg/dL for nonfasted, morning samples (i.e. before 12 pm) and < 57.0 μg/dL for non-fasted, afternoon samples (i.e. after 12 pm) for 6–59 months young children^31^.

Serum zinc concentration was determined by Microwave Plasma Atomic Emission Spectrometry (MP-AES) (Agilent Technologies; model 4200; series MY15260009/G8003A; Nitrogen generator: model 4107, series CN15270004; Autosampler: model SPS3, MY15250009/G8480A Series; Vapor generator accessory: VGA 77, series MY15260005/G8475A) at the Institute of Nutrition of Central American and Panama (INCAP) (Guatemala City, Guatemala)^35^.

### Statistical analysis

All analyses were performed using Stata 15 (StataCorp LLC, Texas, USA)^37^. The reported values (percentage) were weighted by sample weights and 95% confidence intervals (CIs) were included for all the point prevalence considering the cluster sampling design. Logistic regression was used to assess unadjusted and adjusted odds ratio (AOR) and the 95% CIs of ORs. The bivariate association of STHs was assessed with the covariates: age, gender (children only), marital status (women only), lactation (women only), given birth in last five years (women only), level of education (women only), ecological zone (mountain, hills and terai), place of residence (rural and urban), and household wealth quintile (wealth quintile was calculated using household assets such as materials used for flooring, roofing, external walls, sources of drinking water, and possession of households amenities). Principal component analysis was used to calculate the wealth index and further ranked as the wealth quintile, caste/ethnicity (*Brahmin* or *Chettri*, Hill *Dalit*, Terai *Dalit*, *Newar*, Hill *Janajati*, Terai *Janajati*, Other Terai Castes, Muslims and Others), stunting (children only), wasting (children only), underweight (children only), BMI (women only), anemia (Hb < 12.0 g/dL), serum ferritin, risk of folate deficiency and zinc deficiency. Non-normally distributed variables were log transformed. Only significant association observed in bivariate logistic regression were included in the multivariate logistic regression model. Multicollinearity among the predictor variables were checked for variance inflation factors (VIFs). The predictors with the VIFs < 2.0 were only included in the multivariate logistic regression model. P < 0.05 was considered to be statistically significant.

### Ethical approval and consent to participate

The ethical approval to conduct the survey was approved by the Ethical Review Board (ERB) of Nepal Health Research Council (NHRC) (Reg. No.: 201/2015), Ministry of Health and Population, Government of Nepal. Well informed written consent was obtained from all study participants before enrolled in the study. Parental consent was acquired for the participants of < 18 years to participate in the study at the time of survey in local community, eligible and interested adolescents parents’ written consent was ascertained individually. This study was conducted in accordance with the National Ethical Guidelines for Health Research in Nepal 2019 and the Declaration of Helsinki.

## Results

### Children aged 6–59 months

**Figure 1:**
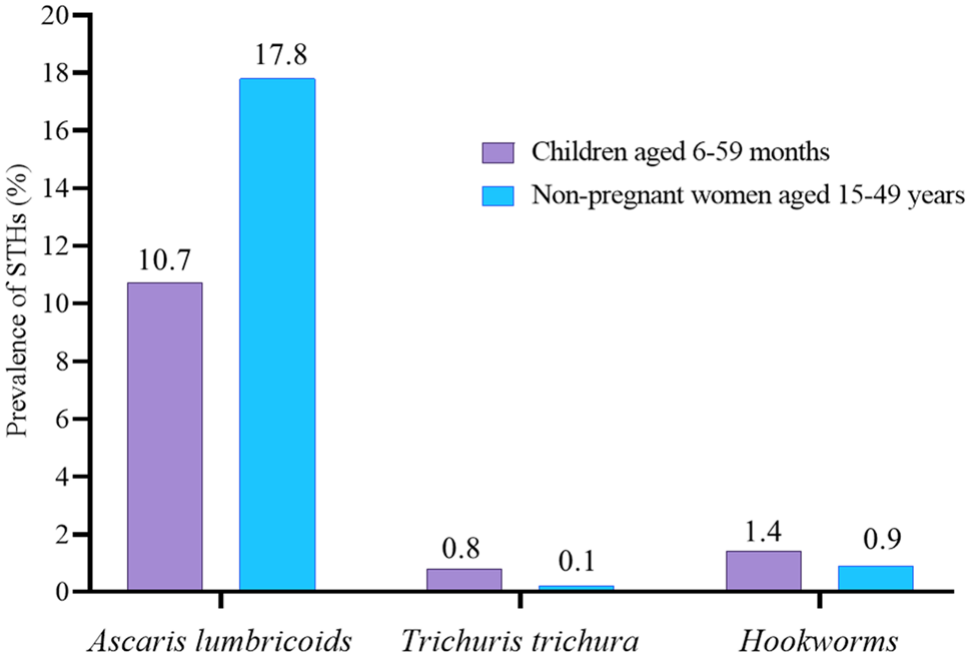
Prevalence of soil transmitted helminths (STHs) among children aged 6–59 months and non-pregnant women aged 15–49 years, Nepal National Micronutrient Status Survey, 2016.

Overall, 12% (95% CI 10.2, 14.0) children aged 6–59 months had STHs (Table 2). The prevalence of *A. lumbricoides* was 10.7%, followed by hookworms and *T. trichura* were 1.4%, and 0.8%, respectively (Fig. 1).

**Table 2:** Socio-demographic and health characteristics of children aged 6–59 months by status of soil transmitted helminths, Nepal National Micronutrient Status Survey, Nepal, 2016.

Socio-demographic and health characteristics	Any soil transmitted helminths^h^ (N = 186, 11.9% [95% CI 10.2, 14.0]		Not any soil transmitted helminths (N = 1349, 88.1% [95% CI 86.0, 89.8]		Total (N = 1535)	
	*n*		*n*		N	
*Socio-demographic characteristics*						
Age (months)	186	35.9 (33.6, 38.2)	1349	33.2 (32.05, 34.4)	1535	33.5 (32.5, 34.6)
Sex (%)						
Male	103	59.0 (48.9, 68.4)	682	54.0 (51.0, 57.1)	785	54.6 (51.6, 57.6)
Female	83	41.0 (31.6, 51.1)	667	46.0 (42.9, 49.0)	750	45.4 (42.4, 48.4)
Rurality (%)						
Rural	159	86.8 (76.1, 93.2)	1172	87.7 (80.7, 92.3)	1331	87.6 (80.6, 92.3)
Urban	27	13.2 (6.8, 23.9)	177	12.3 (7.7, 19.3)	204	12.4 (7.7, 19.4)
Ecological zone (%)						
Mountain	28	8.1 (5.5, 11.6)	225	8.0 (6.1, 10.4)	253	8.0 (6.1, 10.4)
Hill	74	44.7 (33.6, 56.4)	576	42.9 (37.3, 48.6)	650	43.1 (37.5,48.9)
Terai	84	47.2 (36.8, 57.9)	548	49.1 (43.3, 54.9)	632	48.9 (43.1, 54.7)
Household wealth quintile (%)						
Poorest	48	21.5 (14.7, 30.1)	377	20.9 (16.7, 25.9)	425	20.9 (16.8, 25.8)
Poorer	29	18.6 (12.3, 27.3)	296	20.5 (16.9, 24.8)	325	20.3 (16.8, 24.3)
Middle	33	20.5 (13.6, 29.7)	234	19.1 (15.4, 23.6)	267	19.3 (15.8, 23.3)
Richer	48	23.5 (16.0, 33.2)	234	19.8 (16.3, 23.6)	282	20.1 (17.0, 23.7)
Richest	28	15.9 (9.0, 26.5)	208	19.7 (14.5, 26.3)	236	19.4 (14.3, 25.6)
Ethnicity (%)						
*Brahmin* or *Chettri*	70	27.5 (20.4, 35.9)	477	31.4 (26.2, 37.1)	547	30.9 (25.9, 36.4)
Hill *Dalit*	19	6.5 (3.6, 11.5)	228	11.8 (9.1, 15.1)	247	11.1 (8.5, 14.4)
Terai *Dalit*	17	11.6 (5.7, 21.9)	64	6.6 (4.0, 10.8)	81	7.2 (4.3, 11.7)
*Newar*	9	5.7 (2.2, 14.0)	38	3.3 (1.9, 5.7)	47	3.6 (2.2, 5.9)
Hill *Janajati*	38	24.1 (14.5, 37.2)	309	23.5 (18.4, 29.6)	347	23.6 (18.2, 29.9)
Terai *Janajati*	11	4.8 (2.3, 14.3)	88	5.6 (3.8, 8.1)	99	5.5 (3.8, 7.9)
Other Terai Castes	12	13.0 (5.8, 26.9)	104	14.1 (8.2, 23.1)	116	14.0 (8.1, 23.0)
Muslims	9	6.5 (2.8, 14.3)	40	3.6 (1.9, 6.8)	49	4.0 (2.1, 7.3)
Others	1		1		2	
Hemoglobin^a^	182	11.7 (11.5, 11.9)	1312	11.9 (11.8, 12.0)	1494	11.8 (11.7, 11.9)
Anemia (%)^b^	36	25.3 (18.3, 33.8)	231	18.4 (15.5, 21.7)	267	19.2 (16.3, 22.6)
Anthropometry (%)						
Stunting	80	42.7 (33.6, 52.3)	501	35.3 (31.1, 39.7)	581	36.2 (32.1, 40.5)
Wasting	20	12.1 (6.5, 21.4)	146	12.0 (9.6, 14.9)	166	12.0 (9.7, 14.9)
Underweight	57	31.9 (22.8, 42.7)	404	29.8 (26.0, 33.9)	461	30.0 (26.2, 34.2)
Two-week morbidity recall (%)						
Fever	65	34.3 (25.9, 43.7)	492	36.1 (32.8, 39.6)	557	35.9 (32.6, 39.3)
Cough	72	37.8 (28.8, 47.7)	503	37.8 (34.3, 41.4)	575	37.8 (34.3, 41.4)
Diarrhoea	36	19.2 (13.3, 26.9)	260	19.8 (17.1, 22.9)	296	19.7 (17.1, 22.7)
CRP	183	2.02 (1.15, 2.89)	1311	1.94 (1.66, 2.23)	1494	1.95 (1.70, 2.21)
AGP	183	0.83 (0.73, 0.95)	1311	0.87 (0.83, 0.91)	1494	0.87 (0.83, 0.90)
Malaria (%)	0		0			
*Helicobacter pylori* (%)	42	23.7 (16.1, 33.6)	259	19.2 (15.8, 23.0)	301	19.7 (16.6, 23.2)
Received deworming^c^ (%)	31	82.0 (73.6, 88.2)	1101	80.5 (77.5, 83.2)	1132	80.7 (77.8, 83.3)
Micronutrient status						
Serum ferritin	183	24.69 (20.79, 28.59)	1311	26.33 (24.52, 28.14)	1494	26.1 (24.4, 27.8)
Iron deficiency by ferritin (%)^d^	40	21.9 (16.3, 28.7)	279	23.0 (19.7, 26.8)	319	22.9 (19.9, 26.2)
Serum RBP	183	1.00 (0.95, 1.06)	1131	1.02 (1.00, 1.03)	1314	1.01 (0.99, 1.03)
Vitamin A deficiency(%)^e^	1	1.8 (0.2, 12.7)	16	4.6 (2.7, 7.8)	17	4.3 (2.5, 7.2)
RBC folate	182	698.96 (644.25, 753.66)	1305	710.04 (680.26, 739.83)	1487	708.69 (680.16, 737.23)
Risk of folate deficiency (%)^f^	8	6.1 (2.4, 14.4)	68	5.7 (3.8, 8.6)	76	5.8 (3.8, 8.7)
Zinc	183	84.2 (75.2, 93.1)	1308	89.5 (86.3, 92.7)	1491	88.8 (85.6, 92.0)
Zinc deficiency (%)^g^	52	28.4 (20.6, 37.7)	285	20.6 (17.5, 24.1)	337	21.6 (18.6, 24.9)

Ns are unweighted. Values presented are mean (95% CI) or percent (95% CI).

All estimates account for weighting and complex sampling design.

AGP, ɑ-1 acid glycoprotein; CI, confidence interval; CRP, C-reactive protein; RBC, red blood cell; RBP, retinol binding protein.

^a^Haemoglobin adjusted for altitude and smoking^28^.

^b^Anaemia defined as altitude-and smoking-adjusted Hb < 12.0 g/dL^28^.

^c^Receiving deworming during the 6 months preceding the survey.

^d^Iron deficiency defined as inflammation-adjusted serum ferritin < 15.0 μg/L^28^.

^e^Vitamin A deficiency was defined as RBP < 0.64 μmol/L^28^.

^f^Folate cutoff based on the risk of megaloblastic anaemia defined as RBC folate < 305.0 nmol/L^28^.

^g^Zinc deficiency defined as serum zinc < 65.0 μg/dL for nonfasted, morning (i.e. before 12 pm) samples and < 57.0 μg/dL for non-fasted, afternoon (i.e. after 12 p.m.) samples, inflammation adjusted^28^.

^h^Soil-transmitted helminths including hookworm, *T. trichura*, and *A. lumbricodes.*

**Figure 2:**
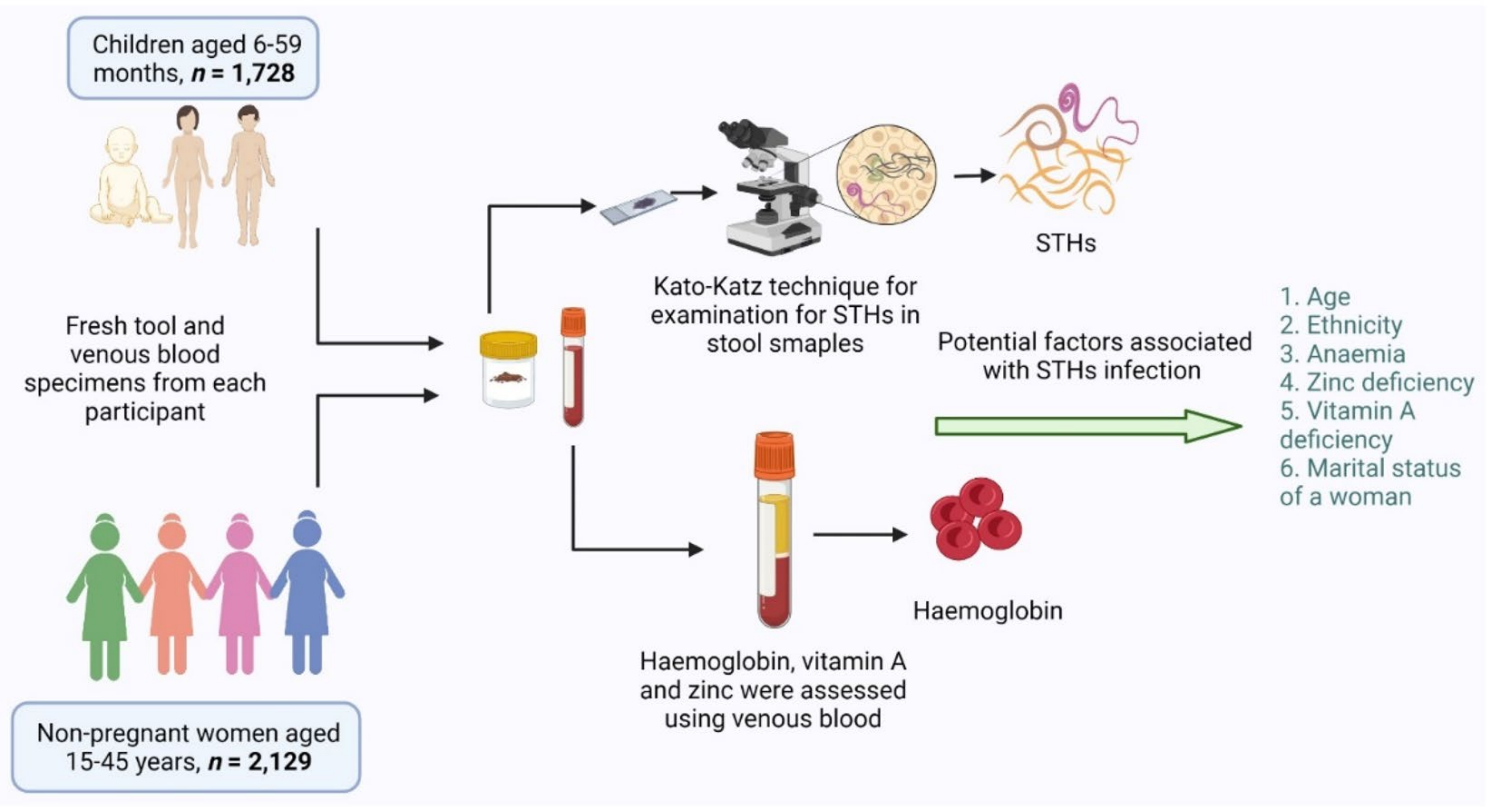
Graphical summary of the study design and major findings, figure was created with BioRender.com.

Results of bivariate and multivariate analysis are presented on Table 3. In multivariate model, age ethnicity, anemia and zinc deficiency were associated with the occurrence of STHs. The likelihood of occurrence of STHs increases significantly with per month increase in age (AOR 1.02; 95% CI 1.00, 1.03). By ethnicity, compared to *Brahmin*/*Chettri*, the significantly higher odds of occurrence of STHs was observed among Terai *Dalits* (AOR 1.97; 95% CI 1.07, 3.63). Likewise, the significantly higher likelihood of occurrence of STHs was observed among the anemic 6–59-month children compared to non-anemic (AOR 1.74; 95% CI 1.12, 2.73). Similarly, those with zinc deficiency, the significantly higher odds of occurrence of STHs was observed compared to 6–59 months children who had normal zinc level (Fig. 2). All the non-significant predictors are presented in the Annex Table 1 (Supplementary Material).

**Table 3:** Predictors of soil transmitted helminths among children aged 6–59 months, Nepal National Micronutrient Status Survey, Nepal, 2016.

Potential predictors	Unadjusted odds ratio (95% CI)	Adjusted odds ratio (95% CI)	*P* value
**Age in months**	1.01 (1.00, 1.02)	1.02 (1.00, 1.03)	0.006
**Ethnicity**			
*Brahmin* or *Chettri*	1	1	
Hill *Dalit*	0.63 (0.34, 1.17)	0.61 (0.32, 1.16)	0.132
Terai *Dalit*	2.00 (1.07, 3.75)	1.97 (1.07, 3.63)	0.029
*Newar*	1.96 (0.63, 6.14)	2.22 (0.70, 7.06)	0.177
Hill *Janajati*	1.17 (0.72, 1.90)	1.13 (0.71, 1.80)	0.608
Terai *Janajati*	0.97 (0.45, 2.10)	0.85 (0.38, 1.87)	0.684
Other Terai Castes	1.05 (0.52, 2.16)	0.92 (0.43, 1.99)	0.833
Muslims	2.02 (0.92, 4.44)	1.88 (0.89, 3.97)	0.098
Others	8.43 (5.88, 12.08)	6.31 (4.08, 9.78)	< 0.001
**Anemia**			
No	1	1	
Yes	1.50 (1.02, 2.21)	1.74 (1.12, 2.73)	0.015
**Zinc Deficiency**			
No	1	1	
Yes	1.52 (0.96, 2.41)	1.61 (1.01, 2.54)	0.043

Estimates are unadjusted odds ratios and adjusted odds ratios with 95% confidence intervals from logistic regression models, accounting for weighting and complex sampling design.

Anaemia was defined as altitude- and smoking- adjusted Hb < 12.0 g/dL^28^.

^g^Zinc deficiency defined as serum zinc < 65.0 μg/dL for nonfasted, morning (i. e. before 12 pm) samples and < 57.0 μg/dL for non-fasted, afternoon (i. e. after 12 p.m.) samples, inflammation adjusted^28^.

### Non pregnant women aged 15–49 years

Overall, 19% (95% CI 16.0, 21.4) non-pregnant women aged 15–49 years had STHs (Table 4). The prevalence of *A. lumbricoids* was 17.8%, whereas the hookworms and *T. trichura* were < 1% each (Fig. 1).

**Table 4:** Socio-demographic and health characteristics of non-pregnant women aged 15–49 years by status of soil transmitted helminths, Nepal National Micronutrient Status Survey, Nepal, 2016.

Socio-demographic and health characteristics	Any soil transmitted helminths^h^ (N = 355, 18.6% [95% CI 16.0, 21.4])		Not any soil transmitted helminths (N = 1657, 81.4% [95% CI 78.6, 84.0])		Total (N = 2012)	
	N		*N*		*n*	
**Socio-demographic characteristics**						
Age group (%)						
15–29 years	188	53.6 (47.3, 59.8)	828	49.2 (46.1, 52.3)	1016	50.0 (47.4, 52.6)
30–49 years	167	46.4 (40.2, 52.7)	829	50.8 (47.7, 53.9)	996	50.0 (47.4, 52.6)
Lactating (%)	101	30.3 (23.7, 37.8)	455	24.0 (21.4, 26.8)	556	25.2 (22.5, 28.0)
Gave birth in last 5 years (%)	146	43.5 (36.1, 51.1)	629	35.0 (32.1, 38.1)	775	36.6 (33.6, 39.7)
Married/cohabitating (%)	320	90.5 (86.2, 93.5)	1384	83.3 (81.0, 85.8)	1704	84.8 (82.4, 86.9)
Rurality (%)						
Rural	290	85.1 (75.0, 91.6)	1440	86.6 (79.2, 91.7)	1730	86.3 (79.2, 91.3)
Urban	65	14.9 (8.4, 25.0)	217	13.4 (8.3, 20.8)	282	13.7 (8.7, 20.8)
Ecological zone (%)						
Mountain	42	5.2 (3.7, 7.5)	299	6.7 (5.3, 8.4)	341	6.4 (5.1, 8.1)
Hill	155	49.9 (41.4, 58.4)	707	43.6 (38.5, 48.8)	862	44.8 (39.9, 49.7)
Terai	158	44.9 (36.6, 53.4)	651	49.7 (44.5, 54.9)	809	48.8 (44.0, 53.7)
Household wealth quintile (%)						
Poorest	58	12.9 (9.0, 18.3)	393	15.9 (12.7, 19.8)	451	15.4 (12.4, 19.0)
Poorer	69	17.9 (13.6, 23.1)	361	19.4 (15.9, 23.5)	430	19.2 (15.9, 22.9)
Middle	65	20.2 (14.7, 27.3)	323	20.0 (16.7, 23.6)	388	20.0 (17.0, 23.4)
Richer	76	21.2 (15.0, 28.9)	292	19.6 (16.3, 23.3)	368	19.9 (16.7, 23.5)
Richest	87	27.8 (18.9, 38.9)	288	25.1 (18.8, 32.6)	375	25.5 (19.4, 33.0)
Ethnicity (%)						
*Brahmin* or *Chettri*	151	38.9 (31.0, 47.4)	664	37.5 (32.0, 43.5)	815	37.8 (32.4, 43.5)
Hill *Dalit*	45	9.1 (5.6, 14.5)	198	8.7 (6.7, 11.3)	243	8.8 (6.8, 11.3)
Terai *Dalit*	19	7.5 (2.9, 18.1)	66	6.2 (3.7, 10.4)	85	6.5 (3.8, 10.9)
*Newar*	19	7.1 (4.0, 12.3)	50	4.4 (2.5, 7.5)	69	4.9 (2.9, 8.1)
Hill *Janajati*	67	19.0 (14.1, 25.0)	406	22.6 (18.0, 28.0)	473	21.9 (17.7, 26.9)
Terai *Janajati*	21	4.7 (2.5, 8.6)	160	10.4 (7.0, 15.1)	181	9.3 (6.4, 13.5)
Other Terai Castes	20	10.6 (4.4, 23.1)	90	8.6 (5.1, 14.2)	110	9.0 (5.2, 14.9)
Muslims	13	3.2 (1.2, 8.3)	21	1.4 (0.7, 2.8)	34	1.8 (0.9, 3.6)
Others	0		2		2	
Level of education (%)						
Never attended school	110	30.9 (23.9, 39.0)	575	32.1 (27.9, 36.5)	685	31.8 (27.9, 36.1)
Primary	70	20.2 (15.7, 25.5)	250	14.8 (12.8. 17.1)	320	15.8 (14.0, 17.9)
Some secondary	123	34.1 (27.0, 42.0)	610	38.1 (34.7, 41.6)	733	37.4 (34.0, 40.8)
Higher	52	14.8 (11.2, 19.5)	222	15.0 (12.5, 18.0)	274	15.0 (12.7, 17.6)
Hemoglobin^a^	354	13.07 (12.85, 13.28)	1655	12.95 (12.84, 13.06)	2009	12.98 (12.87, 13.08)
Anemia (%)^b^	45	14.6 (10.4, 20.1)	286	19.9 (17.1, 23.1)	331	18.9 (16.3, 21.9)
Body Mass Index (%)						
Underweight	41	11.5 (8.5, 15.3)	264	15.4 (13.1, 17.9)	305	14.6 (12.6, 16.9)
Normal weight	233	63.0 (56.2, 69.4)	1035	61.2 (57.8, 64.5)	1268	61.6 (58.5, 64.5)
Overweight/Obese	81	25.5 (19.2, 32.9)	353	23.4 (20.3, 26.9)	434	23.8 (20.8, 27.2)
Two week morbidity recall (%)						
Fever	39	11.0 (7.6, 15.6)	281	14.9 (12.5, 17.7)	320	14.2 (12.1, 16.6)
Cough	58	15.8 (11.6, 21.1)	272	14.6 (12.6, 16.9)	330	14.8 (12.8, 17.1)
Diarrhea	29	9.2 (5.9, 14.0)	161	9.5 (7.7, 11.6)	190	9.4 (7.8, 11.4)
CRP	354	1.42 (0.98, 1.87)	1650	1.45 (1.24, 1.66)	2004	1.44 (1.26, 1.63)
AGP	354	0.61 (0.58, 0.64)	1650	0.60 (0.59, 0.62)	2004	0.60 (0.59, 0.62)
Malaria (%)	0		0		0	
*Helicobacter pylori* (%)	154	42.0 (35.3, 49.0)	648	39.6 (35.2, 44.1)	802	40.0 (36.2, 44.0)
Received deworming (%)^c^	155	34.2 (26.5, 42.9)	783	40.7 (36.2, 45.3)	938	39.5 (35.1, 44.0)
Micronutrient status						
Serum ferritin	354	37.25 (33.84, 40.65)	1650	39.14 (37.10, 41.19)	2004	38.79 (36.95, 40.64)
Iron deficiency by ferritin (%)^d^	59	17.4 (12.3, 24.0)	295	19.2 (16.4, 22.2)	354	18.8 (16.4, 21.5)
Serum RBP	354	1.44 (1.39, 1.49)	1650	1.44 (1.41, 1.46)	2004	1.44 (1.41, 1.46)
Vitamin A deficiency (%)^e^	6	7.1 (2.9, 16.5)	6	1.8 (0.7, 4.4)	12	2.8 (1.5, 5.1)
RBC folate	354	585.32 (555.66, 614.99)	1655	603.12 (570.51, 635.74)	2009	599.82 (571.81, 627.83)
Risk of folate deficiency (%)^f^	32	7.7 (5.0, 11.6)	210	11.2 (9.1, 13.9)	242	12.4 (7.9, 19.0)
Zinc	353	83.60 (79.02, 88.18)	1648	84.15 (81.13, 87.17)	2001	84.05 (81.25, 86.85)
Zinc Deficiency (%)^g^	80	22.6 (16.7, 29.9)	435	25.1 (22.1, 28.3)	515	24.6 (21.6, 27.9)

Ns are unweighted. Values presented are mean (95% CI) or percent (95% CI).

All estimates account for weighting and complex sampling design.

AGP, ɑ-1 acid glycoprotein; BMI, Body Mass Index; CI, confidence interval; CRP, C-reactive protein; RBC, red blood cell; RBP, retinol binding protein.

^a^Haemoglobin adjusted for altitude and smoking^28^.

^b^Anaemia defined as altitude-and smoking-adjusted Hb < 12.0 g/dL^28^.

^c^Receiving deworming during the 6 months preceding the survey.

^d^Iron deficiency defined as inflammation-adjusted serum ferritin < 15.0 μg/L^28^.

^e^Vitamin A deficiency was defined as RBP < 0.64 μmol/L^28^.

^f^Folate cutoff based on the risk of megaloblastic anaemia defined as RBC folate < 305.0 nmol/L^28^.

^g^Zinc deficiency defined as serum zinc < 66.0 μg/dL for nonfasted, morning (i. e. before 12 pm) samples and < 59.0 μg/dL for non-fasted, afternoon (i. e. after 12 p.m.) samples^28^.

^h^Soil-transmitted helminths including hookworm, *Trichuris trichura*, and *Ascaris lumbricodes.*

In multivariate model, compared to unmarried, the significantly lower odds of STHs occurrence was observed among married/cohabitating women (AOR 0.60; 95% CI 0.39, 0.90). Compared to *Brahmin*/*Chettri*, the significantly lower odds of STHs occurrence was observed among Terai *Janajatis* women (AOR 0.45; 95% CI 0.23, 0.88) whereas the higher odds of occurrence was observed among *Newars* (AOR 1.65; 95% CI 1.07, 2.56). Similarly, the higher odds of occurrence of STHs was observed among non-pregnant women aged 15–49 years who had Vitamin A deficiency (AOR 4.72; 95% CI 1.00, 22.40) (Table 5). The wide range of 95% CI might be due to sample size as vitamin A was assessed only in sub sample; 653 of pre-school children and 529 of non-pregnant women. All the non-significant predictors are presented in the Annex Table 2 (Supplementary Material).

**Table 5:** Predictors of soil transmitted helminths among non-pregnant women aged 15–49 years, Nepal National Micronutrient Status Survey, Nepal, 2016.

Potential predictors	Unadjusted odds ratio (95% CI)	Adjusted odds ratio (95% CI)	*P *value
**Gave birth in last 5 years, %**			
No			
Yes	0.70 (0.51, 0.96)	0.78 (0.56, 1.09)	0.141
**Marital status, %**			
Unmarried			
Married/cohabitating	0.54 (0.35, 0.81)	0.60 (0.39, 0.90)	0.015
**Ethnicity**			
*Brahmin* or *Chettri*	1	1	
Hill *Dalit*	1.00 (0.59, 1.71)	0.97 (0.57, 1.66)	0.924
Terai *Dalit*	1.17 (0.47, 2.91)	1.17 (0.48, 2.86)	0.735
*Newar*	1.55 (0.99, 2.44)	1.65 (1.07, 2.56)	0.025
Hill *Janajati*	0.81 (0.56, 1.17)	0.79 (0.55, 1.14)	0.208
Terai *Janajati*	0.43 (0.22, 0.85)	0.45 (0.23, 0.88)	0.019
Other Terai Castes	1.19 (0.52, 2.72)	1.16 (0.51, 2.62)	0.728
Muslims	2.21 (1.12, 4.34)	2.10 (1.05, 4.21)	0.036
Others			
**Vitamin A deficiency**			
No	1	1	
Yes	4.09 (1.05, 15.97)	4.72 (1.00, 22.40)	0.05

Estimates are unadjusted odds ratios and adjusted odds ratios with 95% confidence intervals from logistic regression models, accounting for weighting and complex sampling design.

Vitamin A deficiency was defined as RBP < 0.64 μmol/L^28^.

## Discussion

More than one fourth of the global population is at risk of STHs infection, especially women of reproductive age (WRA), school aged children (SAC), and pre-school aged children (PSAC) are at higher risk^38^ and helminthic parasites are causing a considerable disease and disability in Nepal^39,40^. We evaluated the nationally representative samples (NNMSS-2016) collected from non-pregnant women (15–49 years) and children (6–59 months) covering all three ecological regions and determined the prevalence and associated potential risk factors of STHs infections in Nepal. Earlier cross-sectional studies were more focused on intestinal parasitic infections (IPIs) among young children of a particular area or school^41,42^ so, data on samples from all over Nepal including various age groups is very scanty. To the best of authors knowledge, this is the first study which includes nationwide data along with wider potential risk factors linked with STHs infections among young children and non-pregnant women.

Overall, 12% (95% CI 10.2, 14.0) of children aged 6–59 months had STHs infection where *A. lumbricoides* was the most common helminth (10.7%) followed by *T. trichura* (1.4%), and hookworms (0.8%), respectively. A study conducted among school children (9–12 years) of Bhaktapur, a neighboring district of Kathmandu has reported 27.7% prevalence of intestinal helminth parasites where *A. lumbricoides* (22.6%) was the most detected parasite followed by *T. trichiura* (6.06%)^43,44^ while prevalence was 42.5% among school going children in Kathmandu and *A. lumbricoides* (56.1%) and *T. trichiura* (17.5%) were the most common^19^. The low prevalence of STHs in our study could be due to nationwide deworming programs initiated in 2004 with vitamin A supplement focusing preschool children^45^. A findings from a meta-analyses by Kunwar et al. have observed a sharp decline in prevalence of STHs among school-aged children in Nepal in the last decade^46^. Higher rate (57.2%) of STHs infections was reported among school-aged children (6–12 years) from a village school of Indonesia^47^ where 62.39% of children used to play with dirt/soil each day. Similarly, high prevalence of STHs infections (75.6%) was reported from primary school going children of Utter Pradesh, India and *A. lumbricoides* (69.6%) was the most prevalent where open defecation, no use of soap for hand washing after defecation, and living in mud houses were the major predisposing risk factors associated with helminthic infections^48^. On the other hand, a cross-sectional national study conducted by Ediriweera et al. (2019) in Sri Lanka found only 0.97% of STHIs prevalence among primary school children (5–7 years) where 5946 children from 130 schools were included; while the country’s prevalence of STHIs was 6.9% among school children in 2003^49^ and study highlights the importance of deworming program to reduce the burden of STHs infections in rural areas of Sri Lanka. In multivariate analysis of this study, age, ethnicity, anemia, and zinc deficiency were associated with the occurrence of STHs in children where probability of occurrence of STHs increases significantly with per month growth in age of the below five years children. Adding to this, the significantly higher odds of occurrence of STHs was observed among Terai *Dalits* compared to *Brahmin*/*Chettri* while in a cross-sectional study reported from Terai region of Nepal ethnicity was not significantly associated with the prevalence of geohelminth infection among individuals aged 20–60 years and the study showed prevalence of geohelminth closely associated with unhygienic behaviors and malnutrition^50^. Though there is no existing study focusing STHs among 6–59 months children, but it is plausible to mention that prevalence of geohelminth is high among this group belonging to poor socio-economic status in Nepal. Infection caused by STHs often coexist with micronutrient deficiencies among populations of low-income countries^51^. Status of micronutrient including zinc, hemoglobin, iodine, and vitamin A are associated with higher incidence of STHs infections, especially in children. Anemia and zinc deficiency showed significant association with the occurrence of STHs among children population of this study. Deficiency of certain micronutrients can make a person more susceptible to helminths infections, which can then affect the host's both nutritional and immune status. On the other hand, reduction of nutritional intake due to high intensity infections (many worms) can cause malaise and weakness leading to impaired physical development and fitness of growing age children^52^. Nearly 75% of nations with mild to severe Vitamin A deficiency (VAD) are co-endemic for STHs^53^ presenting two (VAD and STHs) overlapping global health issues so, coadministration of deworming drugs with supplement of vitamin A harness synergistic benefits to the people^54,55^. Pre-school children are at more risk of STHs and they need regular mass medication, termed as ‘preventive chemotherapy (PC)’^56^ and the WHO has recommended periodic large-scale PC when the prevalence of STHs infections surpasses a pre-defined threshold among school age children^57^. Approximately 49% of the WRA from the South East Asia were in need of preventive chemotherapy for STH in 2015^38^. As per the data of WHO Preventive Chemotherapy and Transmission Control (PCT), Nepal has achieved the goal of PC in 2012/2013 with coverage of at least 75% of school aged children at risk. In this study, 82% of children have received deworming drugs. Positive association between *A. lumbricoides* infections and malnutrition among children aged 6–16 years was observed in a national survey conducted in Ecuador^58^. Similarly, zinc deficiency was significantly and negatively correlated with the *Trichuris* infection in Vietnamese school children aged 6–9 years where lower hemoglobin concentration was significantly correlated among children infected by *Trichuris,* hookworm, and multiple helminthic infections compared to uninfected ones^59^. Soil-transmitted helminths feed on host blood, and tissue in addition hookworms can cause intestinal blood loss which leads to anemia. Some of the STHs particularly *T. trichiura* also cause loss of appetite which reduce the nutrients intake and impairs the physical fitness of an individual^60^.

In multivariate analysis, compared to non-pregnant, the significantly lower odds of occurrence of STHs infections observed among married/cohabitating women (AOR 0.60; 95% CI 0.39, 0.90). Similarly, *Newari* ethnicity, and Vitamin A deficiency were predictors of STHs infections among non-pregnant women aged 15–49 years. Overall, 19% non-pregnant women aged 15–49 years had STHs where *A. lumbricoids* (17.8%) was the leading helminth, whereas hookworms and *T. trichura* was detected < 1%. A study conducted among adults (aged > 20, 256 men and 338 women) STHs infection was varied from 3.3% to 51.5% in Nepal where roundworm was the predominant cause of STHs infections. Surprisingly, nutritional status, occupation, and sociodemographic traits were not associated with the STHs infection but personal hygiene was the major risk factor among the enrolled population^61^. Anemia and hookworm infection are common problems among pregnant women so, WHO recommends deworming drugs for reproductive aged women and non-pregnant adolescent girls as a public health intervention in order to reduce the burden of STHs infection where prevalence of any STHs infection is ≥ 20% among reproductive aged women or adolescent girls^48,62^. In this study, STHs infection rate was more (49.9%) among women of Hilly zone compared to Terai (44.9%), and Mountain (5.2%). Similarly, women of rural area were more infected (85.1%) compared to women of urban area (8.4%) and only 34.2% of women have received deworming during the 6 months preceding the survey. In agreement with our study, prevalence of geohelminth infection was higher (76.2%) in rural districts of western Kenya among pregnant women and geohelminth infection was associated with use of polluted drinking water^63^. Similarly, higher prevalence (39%) of STHs infection was observed in a tribal area of southern India where people engaged in agriculture had higher odds (1.68, 95% CI 1.31–2.17, P < 0.001) of hookworm infection^64^. In contrast to current study, STHs infection was more in urban children aged 3–14 years than rural children and reported risk factors were having mothers of aged < 35 years, not wearing shoes, and sewage around residence^65^. A study reported from Nigeria, serum level of iron and vitamin A were significantly lower among STHs pregnant women and school-aged children^66^. Pregnant women may benefit from deworming drugs during their pregnancy, primarily by reducing anemia and a randomized, controlled trial study indicated deworming with mebendazole can be administered safely in antenatal care plan if hookworm infection is endemic^67^. In addition, STHs infection during pregnancy or early age of kids (< 1 year) is associated with poor cognitive outcomes of offspring^68^. Increasing evidence shows that prenatal exposure to STHs infection can impact the susceptibility of progeny, especially to allergy-associated disease^69^.

### Strength and limitations

To the best of our knowledge, this study is the first nationwide epidemiological study to provide a comprehensive understanding of prevalence and determinants of STHs in Nepal. This study has some limitations. Echoing with previous cross-sectional studies, we could not establish causality between the covariates and incidence of STHs. We were unable to account for other potential confounders of STHs infections such as: personal hygiene, sanitation, environmental status, dietary habits, and thus residual confounding could not be excluded as predisposing factors associated with STHs infection in Nepal.

## Conclusion

Despite nationwide deworming program and sanitation awareness, a noticeable prevalence of STHs among 6–59 months children and non-pregnant women was observed in Nepal, particularly children among Terai *Dalits* with anemic and zinc deficiency, and non-pregnant women among *Newari* ethnicity and non-pregnant women with Vitamin A deficiency. The findings of our analysis suggest high-risk populations should be on focus of deworming program, nutrition intervention, and mass administration of preventive chemotherapy along with supplement of vitamin A, iron tablets and Water, Sanitation and Hygiene (WASH) programs for non-pregnant women and young children. There is a need of holistic approach to achieve the progress towards eliminating STHs infection with efficient allocation of resources and developing disease tackling indicators in order to evaluate the dynamics of STHs transmission and infection in Nepal.

## Supplementary Information

Supplementary Information.

## Data Availability

All relevant data are included the paper. The research datasets used and analysed during the current study available from the corresponding author on reasonable request.
