# Supplementary material for: Micronutrients deficiencies and its correlation with the soil-transmitted helminthic infections among children and non-pregnant women in Nepal: findings from Nepal national micronutrient status survey
Source: Sci Rep. 2022 Dec 24;12:22313. doi: 10.1038/s41598-022-24634-3 (PMC9789997; doi:10.1038/s41598-022-24634-3)
Supplement: Supplementary file 1 — Supplementary Information. [file 41598_2022_24634_MOESM1_ESM.pdf]

Annex Table 1: Predictors of soil transmitted helminths among children aged 6 to 59 months, Nepal National Micronutrient Status Survey, Nepal, 2016

**Sociodemographic and health characteristics**	**Unadjusted odds ratio (95% CI)**	**Adjusted odds ratio (95% CI)**	***p*-value**
Sociodemographic characteristics			
Age, months	1.01 (1.00, 1.02)	1.02 (1.00, 1.03)	0.006
Sex			
Male	1		
Female	0.82 (0.54, 1.24)		
Rurality			
Urban	1		
Rural	0.93 (0.53, 1.61)		
Ecological zone (%)			
Hill	1		
Mountain	0.96 (0.50, 1.84)		
Terai	0.92(0.54, 1.54)		
Household wealth quintile			
Poorest			
Poorer	0.88 (0.52, 1.50)		
Middle	1.05 (0.60, 1.83)		
Richer	1.17 (0.63, 2.16)		
Richest	0.79 (0.39, 1.57)		
Ethnicity			
Brahmin or *Chettri*	1	1	
Hill *Dalit*	0.63 (0.34, 1.17)	0.61 (0.32, 1.16)	0.833
Terai *Dalit*	2.00 (1.07, 3.75)	1.97 (1.07, 3.63)	0.132
Newar	1.96 (0.63, 6.14)	2.22 (0.70, 7.06)	0.029
Hill *Janajati*	1.17 (0.72, 1.90)	1.13 (0.71, 1.80)	0.177
Terai *Janajati*	0.97 (0.45, 2.10)	0.85 (0.38, 1.87)	0.608
Other Terai Castes	1.05 (0.52, 2.16)	0.92 (0.43, 1.99)	0.684
Muslims	2.02 (0.92, 4.44)	1.88 (0.89, 3.97)	0.098
Others	8.43 (5.88, 12.08)	6.31 (4.08, 9.78)	<0.001
Hemoglobin	0.88 (0.75, 1.04)		
Anemia			
No	1	1	
Yes	1.50 (1.02, 2.21)	1.74 (1.12, 2.73)	0.015
Anthropometry			
Stunting			
No	1		
Yes	1.36 (0.93, 2.00)		
Wasting			
No	1		
Yes	1.01 (0.51, 1.99)		

Underweight			
No	1		
Yes	1.11 (0.71, 1.73)		
Two weeks morbidity recall			
Fever			
No	1		
Yes	0.92 (0.62, 1.37)		
Cough			
No	1		
Yes	1.00 (0.67, 1.50)		
Diarrhea			
No	1		
Yes	0.96 (0.61, 1.50)		
CRP	1.00 (0.96, 1.04)		
AGP	0.88 (0.54, 1.41)		
Helicobacter pylori			
Negative	1		
Positive	1.31 (0.76, 2.25)		
Received deworming			
Yes	1		
No	0.94 (0.57, 1.56)		
Micronutrient status			
Serum ferritin	1.00 (0.98, 1.01)		
Iron deficiency by ferritin			
No	1		
Yes	0.94 (0.61, 1.44)		
Serum RBP	0.86 (0.39, 1.86)		
Vitamin A deficiency			
No	1		
Yes	0.39 (0.05, 2.89)		
RBC folate	1.00 (0.99, 1.00)		
Risk of folate deficiency			
No	1		
Yes	1.07 (0.45, 2.53)		
Zinc	1.00 (0.99, 1.01)		
Zinc Deficiency			
No	1	1	
Yes	1.52 (0.96, 2.41)	1.61 (1.01, 2.54)	0.043

Note: significant variables in bivariate analysis were included in multivariate analysis

Annex Table 2: Predictors of soil transmitted helminths among non-pregnant women aged 15-49 years, Nepal National Micronutrient Status Survey, Nepal, 2016

Sociodemographic and health characteristics		Unadjusted odds ratio (95% CI)	Adjusted odds ratio (95% CI)	*p*-value
Sociodemographic characteristics				
Age group				
	15-29 years	1		
	30-49 years	0.84 (0.62, 1.13)		
Lactating				
	No	1		
	Yes	1.37 (0.97, 1.94)		
Gave birth in last 5 years				
	No	1		
	Yes	0.70 (0.51, 0.96)	0.78 (0.56, 1.09)	0.141
Married/cohabitating				
	No			
	Yes	0.54 (0.35, 0.81)	0.60 (0.39, 0.90)	0.015
Place of residence				
	Rural	1		
	Urban	0.88 (0.50-1.55)		
Ecological zone				
	Hill	1		
	Mountain	0.69 (0.41, 1.14)		
	Terai	0.79 (0.50, 1.24)		
Household wealth quintile				
	Poorest	1		
	Poorer	1.13(0.74, 1.75)		
	Middle	1.25 (0.75, 2.09)		
	Richer	1.33 (0.81, 2.19)		
	Richest	1.36 (0.81, 2.29)		
Ethnicity				
	Brahmin or *Chettri*	1	1	
	Hill *Dalit*	1.00 (0.59, 1.71)	0.97 (0.57, 1.66)	0.924
	Terai *Dalit*	1.17 (0.47, 2.91)	1.17 (0.48, 2.86)	0.735
	Newar	1.55 (0.99, 2.44)	1.65 (1.07, 2.56)	0.025
	Hill *Janajati*	0.81 (0.56, 1.17)	0.79 (0.55, 1.14)	0.208
	Terai *Janajati*	0.43 (0.22, 0.85)	0.45 (0.23, 0.88)	0.019
	Other Terai Castes	1.19 (0.52, 2.72)	1.16 (0.51, 2.62)	0.728
	Muslims	2.21 (1.12, 4.34)	2.10 (1.05, 4.21)	0.036
	Others			
Level of education				
	Never attended school	1		
	Primary	1.41 (0.96, 2.06)		

	Some secondary	0.93 (0.61, 1.41)		
	Higher	1.02 (0.62, 1.68)		
Hemoglobin		1.07 (0.94, 1.21)		
	Anemia	0.69 (0.46, 1.03)		
BMI				
	Underweight			
	Normal weight	1.38 (0.94, 2.01)		
	Overweight/Obese	1.45 (0.91, 2.31)		
Two week morbidity recall				
Fever				
	Yes	1		
	No	1.42 (0.91, 2.22)		
Cough				
	Yes	1		
	No	0.91 (0.64, 1.30)		
Diarrhea				
	Yes	1		
	No	1.03 (0.62, 1.71)		
CRP		1.00 (0.97, 1.03)		
AGP		1.20 (.70, 2.06)		
Malaria				
Helicobacter pylori				
	Negative	1		
	Positive	1.11 (0.80, 1.54)		
Received deworming				
	Yes	1		
	No	1.32 (0.92, 1.89)		
Micronutrient status				
Serum ferritin		1.00 (0.99, 1.01)		
Iron deficiency by ferritin				
	No	1		
	Yes	0.89 (0.56, 1.40)		
Serum RBP		1.03 (0.75, 1.40)		
Vitamin A deficiency				
	No	1	1	
	Yes	4.09 (1.05, 15.97)	4.72 (1.00, 22.40)	0.05
RBC folate		1.00 (0.99, 1.00)		
Risk of folate deficiency				
	No	1		
	Yes	0.66 (0.41, 1.05)		
Zinc		1.00 (0.99, 1.01)		
Zinc Deficiency				
	No	1		
	Yes	0.87 (0.61, 1.26)		

Note: significant variables in bivariate analysis were included in multivariate analysis.
